# Retropharyngeal Abscess in Children

**Published:** 1908-10-03

**Authors:** 


### Diseases of Children.

### RETROPHARYNGEAL ABSCESS IN CHILDREN.

Although the treatment of retropharyngeal abscess is chiefly surgical, the condition is one which merits the attention of the physician on account of its liability to be forgotten in the differential diagnosis of a case of acute dyspnœa in an infant. The three conditions for which it may be mistaken are laryngismus stridulus, acute catarrhal laryngitis, and laryngeal diphtheria. The reason why it is so important not to mistake the diagnosis is that, whereas the dyspnœa might be such as to require intubation of the larynx or tracheotomy if the condition were diphtheritic, the procedure needed for a postpharyngeal abscess is incision and drainage.

It is essential that in every case of supposed laryngeal diphtheria a finger should be inserted into the child's mouth, and the pharyngeal wall gently but quickly palpated; for palpation is the only sure way of detecting a retropharyngeal abscess. It is hardly ever visible from the mouth; first of all because of the struggles of the child, and of the spasmodic movements of the parts at the back of the buccal cavity; secondly, because the soft palate or enlarged tonsils often come in the way. The finger, on the other hand, can detect the soft fluctuating mass without much difficulty.

It is essential that the palpation should be gentle, lest the abscess should be suddenly ruptured; in which case its contents would almost certainly spout into the larynx, and might cause death from suffocation—the very thing that it is the object of the lateral opening into the abscess cavity from the side of the neck to avoid.

The cause of the acute retropharyngeal abscess is very seldom spinal caries; it is almost always lymphatic infection from the tonsils, fauces, or posterior nares. The lymphatics of the retropharyngeal region form networks on either side, and terminate in small glands located close to the middle line on each side between the pharynx and the aponeurosis covering the prevertebral muscles. These glands exist only in the infant, disappearing soon after the third year just as the thymus gland does; and this agrees with the clinical fact that acute retropharyngeal abscess, simulating laryngeal diphtheria, occurs only in quite small children under four years of age, and most of all in infants. The condition is related to such things as enlarged tonsils and adenoids; mouth-breathing and dirty surroundings strongly predispose to all three. Indeed, the necessity for keeping the possibility of retropharyngeal abscess in mind is much greater in the poorer class of patient.

The last point holds good for another reason also. This is the following:—Although acute laryngeal dyspnœa is the most alarming of the symptoms to which retropharyngeal abscess gives rise, it is by no means the earliest one. Better-class patients would seek advice before the acute dyspnœa was obvious, on account of the snoring character of the breathing, especially during sleep, the loss of appetite, the restlessness, and the tenderness in one side of the neck, which come on gradually and precede the acute dyspnœa by some days. It is in cases where the infant has already been sickly or ailing for some time, or where it is recovering from some other ailment, that these earlier symptoms fail to attract sufficient attention.

In many cases of retropharyngeal abscess a diffuse swelling is visible on one or both sides of the neck, but a sure diagnosis can only be made by examination of the pharynx with the finger.

